# Supplementary material for: Novel Driver Strength Index highlights important cancer genes in TCGA PanCanAtlas patients
Source: PeerJ. 2022 Aug 11;10:e13860. doi: 10.7717/peerj.13860 (PMC9375969; doi:10.7717/peerj.13860)
Supplement: Supplemental Information 2 [file peerj-10-13860-s002.zip › COHORTS/genes plots/cohorts/2021_8_16_14_9_distribution_cohorts_PAAD.pdf]

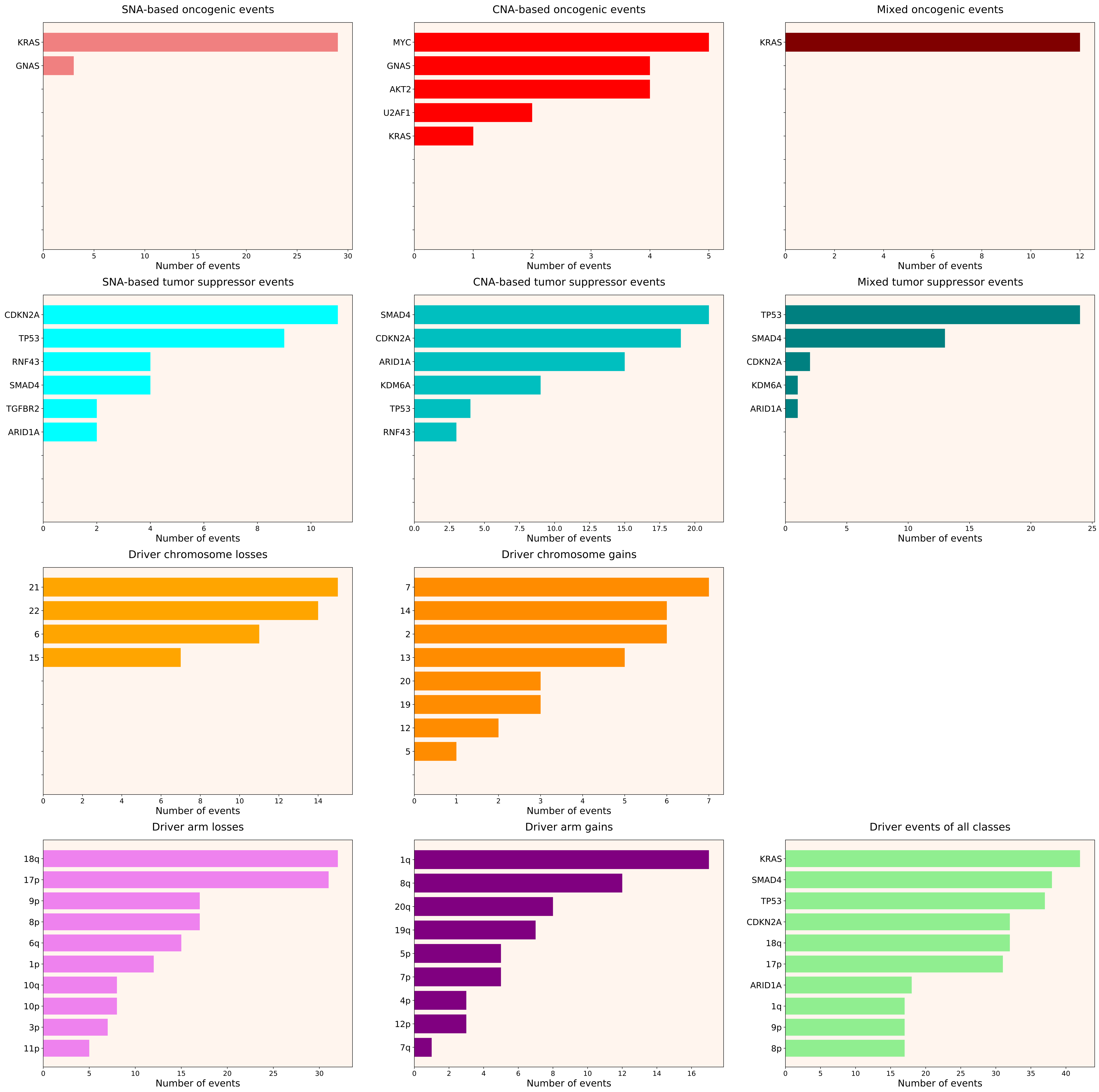
SNA-based oncogenic events
KRAS
GNAS
0 5 10 15 20 25 30
Number of events
CNA-based oncogenic events
MYC
GNAS
AKT2
U2AF1
KRAS
0 1 2 3 4 5
Number of events
Mixed oncogenic events
KRAS
0 2 4 6 8 10 12
Number of events
SNA-based tumor suppressor events
CDKN2A
TP53
RNF43
SMAD4
TGFBR2
ARID1A
0 2 4 6 8 10
Number of events
CNA-based tumor suppressor events
SMAD4
CDKN2A
ARID1A
KDM6A
TP53
RNF43
0.0 2.5 5.0 7.5 10.0 12.5 15.0 17.5 20.0
Number of events
Mixed tumor suppressor events
TP53
SMAD4
CDKN2A
KDM6A
ARID1A
0 5 10 15 20 25
Number of events
Driver chromosome losses
21
22
6
15
0 2 4 6 8 10 12 14
Number of events
Driver chromosome gains
7
14
2
13
20
19
12
5
0 1 2 3 4 5 6 7
Number of events
Driver arm losses
18q
17p
9p
8p
6q
1p
10q
10p
3p
11p
0 5 10 15 20 25 30
Number of events
Driver arm gains
1q
8q
20q
19q
5p
7p
4p
12p
7q
0 2 4 6 8 10 12 14 16
Number of events
Driver events of all classes
KRAS
SMAD4
TP53
CDKN2A
18q
17p
ARID1A
1q
9p
8p
0 5 10 15 20 25 30 35 40
Number of events
